# Core-Perception Coupling: Relationships among Core Temperature, the Rating of Perceived Exertion, and Thirst during Moderate Continuous Exercise under Different Hydration Status

**DOI:** 10.5114/jhk/220099

**Published:** 2026-04-02

**Authors:** Karol Skotniczny, Jan Walencik, Szymon Siatkowski, Jakub Chycki

**Affiliations:** 1 Institute of Sport Sciences, The Jerzy Kukuczka Academy of Physical Education in Katowice, Katowice, Poland.

**Keywords:** thermoregulation, perceived exertion, endurance exercise, exercise-induced dehydration, baseline hydration state

## Abstract

Hydration status modulates both exercise performance capacity and exercise tolerance. This study aimed to examine within-trial relationships among core temperature (Tc), ratings of perceived exertion (RPE), and thirst during moderate continuous cycling, and to test whether baseline hydration status would moderate these associations. Thirty trained men (34.7 ± 6.1 years; VO_2_max 48.8 ± 5.8 ml·kg⁻^1^·min⁻^1^) completed up to 120 min of cycling at 50% Wmax under thermoneutral conditions. Participants were randomized to an experimental group receiving structured hydration counseling (EXP, n = 16) or a control group following habitual fluid intake (CON, n = 14). Hydration status was verified via urine specific gravity; Tc (CORE heat-flux sensor), the heart rate, RPE (0–10), and thirst (0¬–10) were recorded every 15 min. Analyses included repeated-measures tests, Spearman correlations, and ANCOVA adjusting for baselines and VO_2_max. Tc rose from rest and tended to plateau by ~60–75 min, whereas RPE and thirst increased throughout. Hydration status predicted higher RPE (partial η^2^ = 0.41, p < 0.001) with Tc not independently significant (p = 0.81); VO_2_max was not a significant predictor, although the effect estimate suggested an inverse association with RPE (p = 0.058). For thirst, hydration status remained significant (partial η^2^ = 0.27–0.50, p ≤ 0.005). Comparing the two groups, the CON group showed greater increases in RPE (ΔRPE mean +0.23 ± 0.05, p < 0.001; ΔRPE peak +0.53 ± 0.10, p < 0.001) and thirst (ΔThirst mean +0.23±0.04, p < 0.001). EXP participants more often completed the 120-min trial (75.0% vs. 28.6%; χ^2^ = 10.57, p = 0.014). Perceptual coupling was strong (ΔRPE–ΔThirst r_s≈0.84), with ΔRPE inversely related to VO_2_max and ΔTc modestly. Starting exercise euhydrated reduces perceived effort and thirst during prolonged, thermoneutral cycling, while Tc per se adds little explanatory power for RPE after adjustment. Simple field markers (thirst, heart rate) can flag emerging strain, particularly in less fit individuals.

## Introduction

During physical exercise, metabolic heat production increases substantially, elevating core body temperature (Tc). When heat production exceeds the body’s capacity for heat dissipation, particularly in warm or humid environments, hyperthermia may develop. Hydration status and Tc are tightly interrelated, and together they may condition subjective exertion and thirst during exercise (Sawka and Pandolf, 1990). Moreover, observational data indicate that hypohydration is widespread across athletic settings, occurring both before and during training sessions and competitive events (Magee et al., 2017). Field observations in soccer further illustrate the practical relevance of pre-exercise hydration. For example, Czech First League players frequently show urine markers consistent with dehydration and self-assessment of hydration does not always align with objective indices (Klimesova et al., 2022). Similarly, during competition, adolescent soccer players may not hydrate sufficiently to match fluid losses (Fernández-Álvarez et al., 2022). Meta-analytic evidence from endurance exercise indicates a clear dose-response association between exercise-induced dehydration and perceived exertion, with perceived exertion increasing by ~0.21 Borg units for each additional 1% loss of body mass; notably, effects tend to become more pronounced when dehydration exceeds ~3% (Deshayes et al., 2022). Consistently with this, hypohydration has been shown to compromise endurance performance during continuous modalities such as cycling, running, and swimming, and evidence further suggests that performance decrements also extend to intermittent and anaerobic exercise contexts (Logan-Sprenger et al., 2012; Périard et al., 2021). The National Athletic Trainers’ Association further notes that a large proportion of athletes, from youth and secondary-school to collegiate and professional levels begin exercise in a hypohydrated state, and that spontaneous (ad libitum) fluid consumption during activity typically compensates for only approximately two-thirds of sweat losses (McDermott et al., 2017). It therefore appears that hydration status may be an important determinant of changes in perceived exertion and thirst during physical activity. In addition to fluid volume, the mineral content and alkalinity of drinking water can influence hydration- and acid-base-related responses. Previous studies on alkaline or bicarbonate-rich mineral water have reported improved urine-based markers and favorable changes in post-exercise acid-base balance, although performance benefits were not observed consistently across exercise models (Chycki et al., 2017, 2018; Chiron et al., 2024).

Progressive increases in Tc intensify cardiovascular and thermoregulatory strain and are consistently associated with higher ratings of perceived exertion, thereby triggering performance decrements (Tucker et al., 2004). As Tc rises, the body initiates sweating for cooling. A rising Tc can also contribute to central fatigue. In line with this, elevated body temperature leads to increased indices of thermal strain and changes in selected physiological responses in both athletes and non-athletes (Pilch et al., 2022). Heat stress has been shown to reduce the central drive during exercise (for example, by altering brain activity and neurotransmitters activity), causing exercise to be perceived harder although at the same intensity (Cao et al., 2022). Moreover, during hyperthermia, cutaneous vasodilation redistributes cardiac output toward the skin, reducing central venous return and stroke volume and necessitating a compensatory tachycardia to sustain cardiac output (Cheuvront et al., 2010). For a given external workload, this augmented cardiovascular strain increases ratings of perceived exertion (RPE) (Flouris and Schlader, 2015).

Thermal stress also biases substrate utilization toward carbohydrates and accelerates skeletal-muscle glycogen depletion, thereby exacerbating metabolic fatigue and further elevating perceived effort. Dehydrating ~2% over 2 h of cycling raised Tc and RPE significantly, even when the workload was unchanged (Logan-Sprenger et al., 2012). In parallel, heat-sensitive afferent pathways and hypothalamic integrative centers transduce rising Tc into central fatigue signals, amplifying the sensation of “strain”. Notably, evidence supports a critical Tc threshold (≈39–40°C in trained individuals) beyond which protective mechanisms precipitate task disengagement and exhaustion, typically accompanied by very high or maximal RPE (González-Alonso et al., 1999).

Exercise thermoregulation, alterations in Tc, subjective perceptions of fatigue, and hydration status should be considered alongside thirst sensation. Thirst constitutes a central, consciously accessible component of total body water (TBW) homeostatic regulation (Greenleaf, 1992). During exercise, progressive sweating—because sweat is typically hypotonic relative to plasma—tends to increase plasma osmolality and serum electrolyte concentration (notably Na⁺ and Cl⁻), thereby stimulating osmosensitive regions in the lamina terminalis (including the OVLT and SFO) and promoting vasopressin (AVP) release along with thirst (Stachenfeld, 2014; Wang et al., 2023). In parallel, sweat-driven reductions in plasma volume engage cardiopulmonary/baroreflex pathways and neurohumoral responses that further support fluid conservation and drinking behavior, complementing osmotic regulation (Stachenfeld, 2008, 2014). In hot environments, particularly when ambient humidity is elevated and evaporative heat loss is constrained, rapid increases in Tc accelerate sweat losses and hypohydration, amplifying both the osmotic (hyperosmolality/electrolyte concentration) and volume-related drives to drink (Armstrong and Kavouras, 2019). Conversely, the rapid ingestion of large volumes of hypotonic fluid (e.g., plain water) can acutely lower plasma osmolality and dilute extracellular Na⁺/Cl⁻, while oropharyngeal and upper gastrointestinal signals may transiently suppress thirst and AVP before systemic equilibration; under some conditions, excessive drinking relative to sweat and urinary losses may therefore contribute to exercise-associated hyponatremia, particularly when AVP remains inappropriately elevated (Bichet, 2018; Hew-Butler et al., 2017; Salata et al., 1987). By contrast, cold exposure attenuates thirst at rest and during exercise, even when individuals are hypohydrated and hyperosmotic, which suggests an association between hyperthermia and thirst perception (Kenefick et al., 2004). Even at the same level of dehydration, a higher Tc amplifies thirst and vasopressin secretion, further promoting fluid intake (Takamata et al., 1995). From the standpoint of prolonged exercise, it is paramount to monitor dynamic changes in Tc, perceived exertion, thirst sensation, and hydration status.

Therefore, this study aimed to noninvasively assess the relationship among Tc, RPE, and thirst sensation during moderate-intensity exercise across varying baseline hydration states. We hypothesized that, within individuals performing moderate intensity exercise, rising core body temperature (Tc) would lead to higher ratings of perceived exertion (RPEs), indicating that fatigue perception is a consequence of increased thermal strain. Secondly, thirst would rise with both Tc and the magnitude of hypohydration (e.g., percentage body-mass loss or pre-exercise urine indices), with each predictor contributing unique explanatory variance to thirst perception. Third, relative to euhydration, hypohydration would augment thermal strain and amplify the positive associations between Tc and both RPE and thirst, consistently with hydration status moderating the impact of Tc on subjective responses.

## Methods

### Participants

Thirty physically active men (n = 30) with an average age of 34.67 ± 6.12, body mass: 80.6 ± 8.9 kg, body height: 179.7 ± 6.5 cm and VO_2max_: 48.8 ± 5.8 ml·kg⁻^1^·min⁻^1^ were enrolled in the study. A purposeful, representative sample was selected according to the following inclusion criteria: age 25–60 years; non-obese (fat mass <30%), absence of acute disease, and provision of written informed consent. Exclusion criteria were uncontrolled arterial hypertension, unstable coronary artery disease, clinically significant cardiac arrhythmias or an implanted pacemaker, hepatic or renal disease, or refusal/withdrawal of consent.

### Measures

The protocol incorporated diagnostic assessments conducted before and during exercise-induced dehydration. These included body composition profiling using dual-energy X-ray absorptiometry (DXA), continuous monitoring of core temperature dynamics, and administration of subjective scales for perceived exertion and thirst. We selected appropriate instruments to examine the associations among baseline hydration status, exercise-induced thermoregulatory changes, and subjective perception of exercise intensity and thirst.

### Body Composition Assessment (DXA)

Body composition was assessed using whole-body dual-energy X-ray absorptiometry (DXA) on a Lunar iDXA Advance device (GE Healthcare, USA). The system employs K-edge filtered X-ray beams at approximately 39 and 71 keV generated at 100 kV. Each participant underwent full-body scans immediately before the dehydration protocol on the cycle ergometer, in a standardized supine position, according to the manufacturer's instructions. The analysis yielded values for body mass (BM, kg), lean body mass (LBM, kg), fat mass (FM, kg), relative fat mass (FM, %), and bone mineral density (BMD, g/cm^2^), extracted from the system software. The radiation burden per whole-body scan was minimal, with an effective dose of about 0.96–1.92 µSv depending on the scan mode (thin/standard vs. thick), remaining well below 10 µSv in all cases; manufacturer specifications indicate an entrance skin dose of roughly 3 µGy.

### Core Body Temperature

Throughout the intervention, core body temperature was monitored continuously using non-invasive CORE sensors (greenTEG AG, Switzerland). The device estimated core body temperature by integrating cutaneous heat-flux and skin-temperature measurements with environmental inputs, which were processed in real time by a proprietary algorithm combining biothermal heat-transfer modeling with machine-learning calibration against reference methods. When affixed to the chest (sternal region) or the lateral torso per manufacturer guidance, the sensor provided second-by-second core temperature estimates. Measurement accuracy was condition-dependent and could be affected by rapid thermal transitions, motion, and local skin perfusion (Daanen et al., 2023). A recent study by Januário et al. (2024) reported acceptable agreement with ingestible telemetric capsules under steady-state conditions, though accuracy could vary with rapid thermal transitions and motion. We therefore interpreted Tc estimates as approximate rather than criterion values, and not interchangeable with gold-standard rectal, esophageal, or gastrointestinal measurements when absolute Tc thresholds were of interest.

Sensors were mounted according to the manufacturer’s instructions at sites enabling reliable acquisition. Data were streamed in real time to a compatible application. Core body temperature was recorded continuously. For subsequent analyses, values were also logged manually every 15 minutes (0′, 15′, 30′, 45′, 60′, 75′, 90′, 105′, 120′).

### Heart Rate (HR)

The heart rate (HR) was measured with a chest-strap monitor (Polar H10, Polar Electro Oy, Kempele, Finland). The elastic strap was adjusted to fit snugly just inferior to the pectoral muscles at the level of the xiphoid process, with the electrode areas moistened per the manufacturer's instructions. The sensor was positioned at the midline, and strap tension was rechecked before each trial. Prior to each test session, battery status was checked, skin contact was verified, and the strap was cleaned. During recording, the operator monitored signal quality indicators, and brief dropouts or motion artifacts were registered in real time. The HR was recorded continuously throughout a 120-min cycling bout. The H10 streamed data was transferred via Bluetooth to a dedicated receiver (tablet/smartphone) running the manufacturer’s application, which time-stamped all samples. A pre-exercise 5-min seated baseline HR was collected to confirm signal stability.

### Subjective Scales (RPE and Thirst)

RPE was assessed using a 10-point Borg scale (1–10) tailored for continuous aerobic exercise. Participants were familiarized with the scale during the qualification visit. Immediately before exercise (0 min) and at 15-min intervals during the 120-min cycling bout (15, 30, 45, 60, 75, 90, 105, 120 min), participants were shown a laminated sheet displaying the integers 0–10, each accompanied by a brief verbal descriptor. Standard anchors were: 0 = no fatigue/extremely easy, 3 = light, 5 = moderate, 7 = hard, 9 = very hard, 10 = maximal fatigue (Borg, 1998). At each time point, participants pointed to or stated the number that best reflected their overall exertion “right now”. The rater recorded the value immediately on the case report form. Instructions emphasized that ratings should reflect global effort, not localized discomfort, and should be made independently of the heart rate.

Subjective thirst was assessed using a numeric rating scale ranging from 0 to 10 with verbal anchors at the same time points (0, 15, 30, 45, 60, 75, 90, 105, 120 min), as previously described in hydration and hypohydration studies (Armstrong and Kavouras, 2019; Engell et al., 1987). The same laminated sheet format was used, with anchors: 0 = not thirsty at all, 3 = slightly thirsty, 5 = moderately thirsty, 7 = very thirsty, 9 = extremely thirsty, 10 = maximally thirsty. Participants indicated the number that best represented their current thirst sensation; responses were recorded immediately. Before testing, participants were instructed to base ratings on perceived mouth dryness and urge to drink, not on habit or expectations. For both scales, the primary outcomes were time-point values across the 120-min trial.

### Nutrition and Hydration Guidelines

Following the qualification stage, participants in the EXP group were given specific hydration instructions. These included a daily fluid intake target of 2.5 l, which is consistent with current guidance for adult men (EFSA Panel on Dietetic Products, Nutrition and Allergies, 2010). Pre exercise fluid strategies were also standardized. Participants were advised to consume 5 to 7 mL per kg body mass four hours before exercise and, if urine color suggested insufficient hydration, to consume an additional 3 to 5 mL per kg two hours before the onset of exercise, consistently with established fluid intake guidance (Sawka et al., 2007). Participants were asked to self-evaluate urine color using a standardized 8-point urine color chart based on the Armstrong scale, where scores of 1 to 3 were considered indicative of adequate hydration and scores of 4 or higher indicated insufficient hydration requiring additional fluid intake (Armstrong, 1994). During exercise, participants were instructed to ingest 200–300 ml of water every 15 minutes (Casa et al., 2000). Post-exercise rehydration was based on body mass loss, with the recommendation to replace 120–150% of the deficit as determined by pre- and post-exercise body weight measurements. These recommendations applied to participants’ training outside the laboratory. During laboratory trials, from the final pre-test weighing until completion of all post-exercise assessments, no beverages were permitted.

Nutritional intake before testing was standardized. Two hours before the trial, participants consumed a breakfast providing ~4.5 kcal/kg of body weight with a macronutrient distribution of 65% carbohydrates, 25% fats, and 15% proteins. They were also required to ingest 3–5 ml/kg of fluids two hours beforehand. No further beverages were permitted until the end of the intervention phase.

### Hydration Assessment

At each study stage, participants’ hydration status was verified by measuring urine specific gravity using a refractometer (Kern ORM 1SU). The device was calibrated with distilled water in accordance with the manufacturer’s instructions. Each participant completed three assessments at a minimum of weekly intervals. Eligibility to commence the experimental intervention was contingent on meeting the hydration assumptions stipulated by the randomized group assignment, EXP or CON.

### Design and Procedures

In accordance with the a priori assumption that most physically active adults are susceptible to chronic dehydration, participants were randomized to an experimental (EXP) or a control (CON) group using the random.org List Randomizer. The EXP group (n = 16) received structured counseling on hydration and post-exercise fluid replacement, whereas the CON group (n = 14) was instructed to maintain habitual fluid-intake practices. Hydration status was assessed at three weekly time points using fasting urine specific gravity (USG), confirming optimal hydration in the EXP group (mean USG < 1.018) and indicating elevated risk of chronic dehydration in the CON group (mean USG > 1.018). All participants were briefed orally and in writing, informed of their right to withdraw at any time, and provided written informed consent. The protocol was approved by the Bioethics Committee for Scientific Research at the Jerzy Kukuczka Academy of Physical Education in Katowice, Katowice, Poland (approval code: 3-X/2023; approval date: 19 October 2023).

The investigation comprised a single series of experimental procedures, preceded by a qualification phase lasting up to 4 weeks. During qualification, participants underwent physician-supervised screening to verify inclusion and exclusion criteria, completed maximal aerobic capacity testing (VO_2__max_), and were monitored for hydration status. VO_2__max_ as determined using a ramp protocol (20 W·min⁻^1^) on an Excalibur Sport cycle ergometer (Lode). The test commenced at 40 W and progressed with a continuous work-rate increase (resistance increment of 0.33 W·s⁻^1^) at a supervised cadence of 70–80 rev·min⁻^1^, continuing to volitional exhaustion or failure to maintain cadence. Reaching VO_2__max_ was verified by a VO_2_ plateau despite further workload increments (ΔVO_2_ < 150 mL·min⁻^1^) and a respiratory exchange ratio (RER) > 1.10. Throughout rest and exercise, the heart rate (HR), minute ventilation (V̇E), oxygen uptake (V̇O_2_), and carbon dioxide output (V̇CO_2_) were recorded continuously using a MetaLyzer 3B-2R metabolic analyzer (Cortex). For subsequent prescription of relative intensity, maximal power output (W_max_) was estimated individually.

Experimental sessions were conducted following the qualification. Participants attended the laboratory from 08:00 in staggered 30-min intervals, with no more than three individuals scheduled per day. Upon waking, all participants ingested 3–5 ml/kg per body mass of table water, and hydration status was verified via urine specific gravity (USG) before testing. The intervention phase continued until the prespecified sample size had been reached (n = 30). During the experimental phase, the controlled task consisted of cycling at 50% W_max_ referenced to the qualification test for 120 min or until 2–3% dehydration based on body mass loss. Exercise was paused every 15 min to quantify sweat loss via nude body mass measurement conducted in a private room. Before each weighing, participants were thoroughly towel-dried to remove residual sweat to minimize measurement error. The exercise was standardized with respect to cadence, which was maintained within the range of 80–90 revolutions per minute. If the target cadence could not be sustained, the effort was terminated and classified as an inability to continue the exercise. Thermoregulatory responses, particularly core body temperature dynamics were monitored and analyzed continuously, with data collected at 15-min intervals for core temperature, ratings of perceived exertion (RPE), and thirst. At each 15-min time point, participants first completed the questionnaire-based ratings while remaining seated on the cycle ergometer, after which they dismounted for nude body mass assessment to quantify sweat loss. The exercise timer was paused during these interruptions, and each break lasted up to 2 min. Exercise took place under thermoneutral laboratory conditions (22°C, 45% relative humidity, air velocity <0.2 m·s⁻^1^; no solar load). To preclude textile-related influences on core temperature kinetics, participants exercised with the upper body uncovered.

### Statistical Analysis

Descriptive statistics for approximately normally distributed variables are presented as mean ± SD and mean with 95% confidence intervals (CI). Non-normally distributed variables are reported as median [IQR] (range). Values missing at 90 minutes were considered *not missing at random* because termination of exercise after a 3% body mass loss introduced a probability of missingness dependent on unobserved higher core temperature, ratings of perceived exertion (RPE), and thirst, indicating a substantial reduction in activity. To minimize bias associated with non-random or random missingness, primary analyses were restricted to the 0–90-min interval, and no data imputation was applied.

Endpoints were analyzed using analysis of covariance (ANCOVA), with the post-exercise score as the dependent variable, the corresponding baseline score as a covariate, and hydration status (EXP vs. CON) as a between-subjects factor. Additional covariates (e.g., VO_2__max_) were included only if baseline testing indicated significant between-group differences, and parsimonious models were preferred. Effect sizes were expressed as partial eta squared (η^2^) with 95% CIs and interpreted according to conventional thresholds (small ≈ 0.01, medium ≈ 0.06, large ≈ 0.14; Cohen, 1988). Within the 15–90-min interval, participant-specific change scores were calculated as first-order differences between consecutive measurements (Δ = a_2_ − a_1_). The arithmetic mean of these change scores was defined as ΔMean, representing the average response magnitude across the analyzed period. The largest individual change score within the same interval was defined as ΔPeak, representing maximal response magnitude. These aggregated outcomes were subsequently subjected to inferential statistical analyses.

Prior to inferential testing, assumptions were verified as follows: normality of residuals using the Shapiro-Wilk test and Q-Q plots, and homogeneity of variance using the Levene’s test. Between-group comparisons were conducted using the Student’s *t*-test for normally distributed variables and the Mann-Whitney U test for non-normally distributed variables. For *t*-tests, effect sizes were reported as Cohen’s *d* with 95% CIs and interpreted using standard benchmarks (small ≈ 0.20, medium ≈ 0.50, large ≈ 0.80; Cohen, 1988).

The effect of time in repeated-measures data was analyzed using the Friedman test, with effect size quantified as Kendall’s W (small ≈ 0.10, medium ≈ 0.30, large ≈ 0.50). Post hoc pairwise comparisons were performed using the Durbin-Conover procedure with Holm’s correction for multiple testing.

With α = 0.05 and group sizes of EXP (n=16) and CON (n = 14), sensitivity analysis (G*Power 3.1; Heinrich-Heine-Universität Düsseldorf, Germany) indicated 80% power to detect moderate-to-large adjusted effects. For typical pre-post correlations (r ≈ 0.50–0.70), the minimum detectable standardized mean difference was estimated at approximately *d* ≈ 0.76–0.92.

For graphical clarity, data points for the EXP group in Figures 2–4 were displayed with a small horizontal offset on the time axis to avoid overlap with the CON group; this offset was applied for visualization purposes only and did not reflect differences in measurement timing.

**Figure 1:**
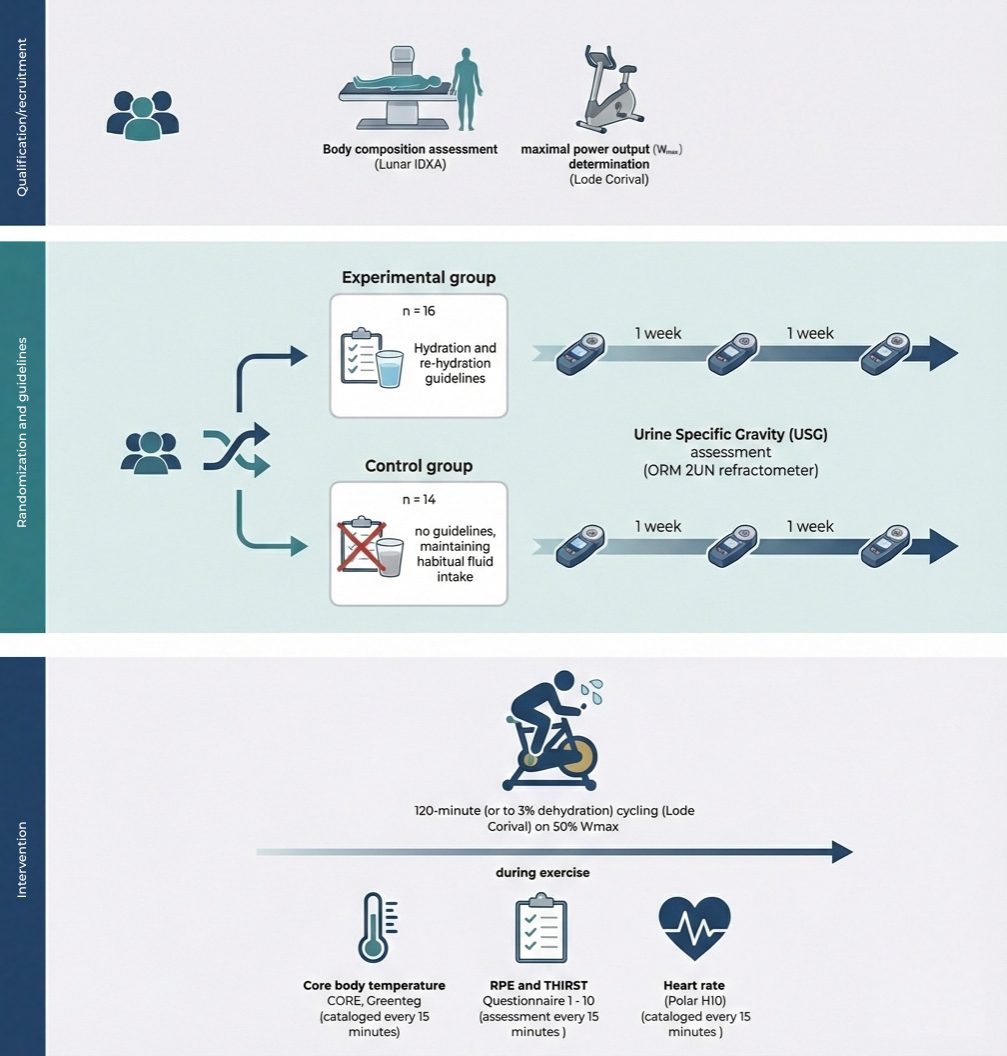
Study flowchart.

**Figure 2:**
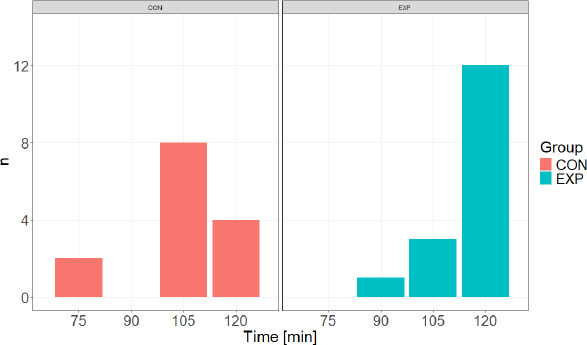
Distribution of exercise duration. CON: Control group, EXP: Experimental group, n: number of participants

**Figure 3:**
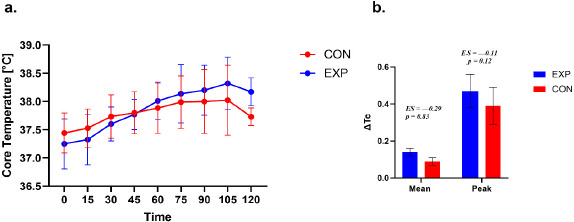
Core body temperature responses during exercise. a. Time course of core body temperature (Tc) during exercise in the experimental (EXP) and control (CON) groups. Values are presented as mean ± SE. For graphical clarity, EXP data points are displayed with a small horizontal offset; this offset is applied for visualization purposes only and does not reflect differences in measurement timing. b. Between-group comparison of Tc response magnitude expressed as ΔMean and ΔPeak. ΔMean represents the average change from baseline across the 15–90 min interval, whereas ΔPeak represents the maximum observed change relative to baseline within the same interval. Statistical analyses were restricted to the 15–90 min interval.

**Figure 4:**
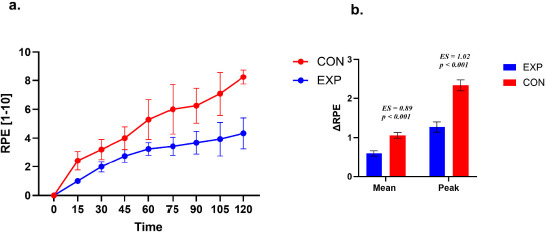
Rating of perceived exertion during exercise. a. Time course of rating of perceived exertion (RPE) during exercise in the experimental (EXP) and control (CON) groups. Values are presented as mean ± SE. EXP data points are displayed with a small horizontal offset to improve visual clarity. b. Between-group comparison of perceptual response magnitude expressed as ΔMean and ΔPeak. ΔMean represents the average change in RPE from baseline across the 15–90 min interval, and ΔPeak represents the highest RPE value observed within the same interval. Statistical analyses were restricted to the 15–90 min interval.

**Figure 5:**
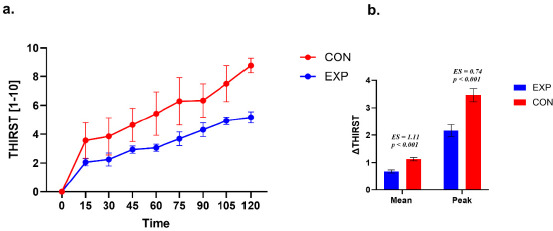
Thirst perception during exercise. a. Time course of thirst perception during exercise in the experimental (EXP) and control (CON) groups. Values are presented as mean ± SE. A small horizontal offset was applied to EXP data points for graphical clarity only. b. Between-group comparison of thirst response magnitude expressed as ΔMean and ΔPeak. ΔMean represents the average change from baseline across the 15–90-min interval, whereas ΔPeak represents the maximum thirst rating observed within this interval. Statistical analyses were restricted to the 15–90-min interval.

**Figure 6:**
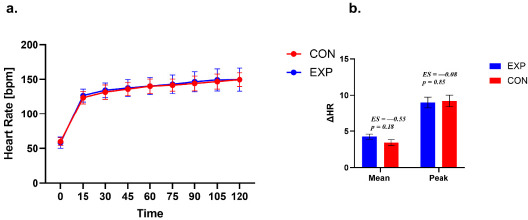
Heart rate during exercise a. Time course of HR during exercise in the experimental (EXP) and control (CON) groups. Values are presented as mean ± SE. A small horizontal offset was applied to EXP data points for graphical clarity only. b. Between-group comparison of thirst response magnitude expressed as ΔMean and ΔPeak. ΔMean represents the average change from baseline across the 15–90-min interval, whereas ΔPeak represents the maximum thirst rating observed within this interval. Statistical analyses were restricted to the 15–90-min interval.

All analyses were performed using Statistica 13.3 (TIBCO Software Inc., Palo Alto, CA, USA), and GraphPad Prism (version 10.6.1; GraphPad Software, LLC, San Diego, CA, USA).. Statistical significance was accepted at a two-tailed α = 0.05.

## Results

Between-group comparisons were performed using independent-samples *t*-tests for normally distributed variables and Mann-Whitney U tests for non-normally distributed variables. Exact *p*-values were reported. Variables showing significant baseline differences were considered for inclusion as covariates in adjusted analyses. Descriptive baseline values are summarized in Table 1.

**Table 1:** Descriptive statistics of baseline measures.

Variable	Total (n = 30)	EXP (n = 16)	CON (n = 14)	Differences between groups	
	M ± SD (±95%CI)			t/Z; *p*	ES
Height [cm]	179.7 ± 6.5 (177.3; 182.1)	180.0 ± 6.9 (176.3; 183.7)	179.3 ± 6.1 (175.7; 182.8)	t = −0.29; *p* = 0.77	*d* = 0.04
Age [y]	34.7 ± 6.1 (32.4; 37.00)	34.3 ± 6.7 (30.7; 37.8)	35.1 ± 5.6 (31.9; 38.4)	t = 0.39; *p* = 0.70	*d* = 0.06
BM [kg]	80.6 ± 8.9 (77.3; 84.0)	81.0 ± 8.8 (76.3; 85.7)	80.3 ± 9.3 (74.9; 85.7)	t = −0.20; *p* = 0.84	*d* = 0.05
BMI [kg·m⁻^2^]	24.9 ± 2.0 (24.2; 25.7)	25.0 ± 2.1 (23.9; 26.1)	24.9 ± 2.1 (23.7; 26.1)	t = −0.84; *p* = 0.94	*d* = 0.05
BSA [m^2^]	2.00 ± 0.14 (1.95; 2.05)	2.01 ± 0.13 (1.93; 2.08)	2.00 ± 0.14 (1.91; 2.08)	t = −0.16; *p* = 0.88	*d* = 0.10
LBM [kg]	64.0 ± 6.9 (61.4; 66.6)	65.6 ± 7.9 (61.4; 69.9)	62.1 ± 5.2 (59.0; 65.1)	Z = −1.35; *p* = 0.18	r_g_ = 0.25
BF [%]	21.2 ± 5.1 (19.3; 23.1)	19.7 ± 4.2 (17.5; 22.0)	22.8 ± 5.7 (19.5; 26.1)	t = 1.74; *p* = 0.10	*d* = 0.32
VO_2max_ [ml·kg⁻^1^·min⁻^1^]	48.8 ± 5.8 (46.6; 51.0)	51.2 ± 6.2 (47.9; 54.5)	46.1 ± 4.0 (43.8; 48.4)	t = −2.66; *p* = 0.01*	*d* = 0.40
USG	1.014 ± 0.008 (1.011; 1.017)	1.009 ± 0.008 (1.005; 1.013)	1.019 ± 0.005 (1.016; 1.022)	Z = 3.25; *p* = 0.001*	r_g_ = 0.59

M: mean; SD: standard deviation; CI: confidence interval; BMI: body mass index, calculated from body mass and stature (kg·m⁻^2^). VO_2__max_: maximal oxygen capacity. Statistically significant differences between groups are indicated by * (p < 0.05)

**Table 2:** Descriptive statistics for repeated measures.

Variable	EXP	CON
	Me ± [IQR] (min–max)	
T_c_ PRE/REST	37.15 [37.09;37.54] (36.03;37.88)	37.33 [37.29;37.57] (36.8;38.28)
T_c_ 15 min	37.21 [37.17;37.60] (36.09;37.99)	37.47 [37.37;37.60] (37.10;38.37)
T_c_ 30 min	37.42 [37.39;37.95] (37.29;38.10)	37.58 [37.52;37.80] (37.19;38.64)
T_c_ 45 min	37.66 [37.57;37.96] (37.43;38.36)	37.68 [37.59;37.86] (37.33;38.90)
T_c_ 60 min	38.01 [37.89;38.08] (37.58;38.99)	37.84 [37.63;37.92] (37.41;39.30)
T_c_ 75 min	38.05 [37.80;38.41] (37.31;39.40)	37.84 [37.73;37.92] (37.64;39.40)
T_c_ 90 min	38.16 [37.89;38.37] (37.76;39.54)	37.90 [37.73;38.04] (37.42;39.63)
T_c_ 105 min	38.33 [38.03;38.58] (37.58;39.55)	37.92 [37.75;38.01] (37.37;39.85)
T_c_ 120 min	38.16 [38.11;38.35] (37.71;38.52)	37.67 [37.63;37.83] (37.63;37.96)
RPE PRE/REST	0.00 [0.00;0.00] (0.00;0.00)	0.00 [0.00;0.00] (0.00;0.00)
RPE 15 min	1.00 [1.00;1.00] (1.00;1.00)	2.50 [2.00;3.00] (1.00;3.00)
RPE 30 min	2.00 [2.00;2.00] (1.00;3.00)	3.00 [3.00;4.00] (2.00;4.00)
RPE 45 min	3.00 [2.50;3.00] (2.00;3.00)	4.00 [3.00;5.00] (3.00;5.00)
RPE 60 min	3.00 [3.00;3.50] (3.00;4.00)	5.00 [5.00;6.00] (3.00;8.00)
RPE 75 min	3.00 [3.00;4.00] (3.00;5.00)	6.00 [5.00;7.00] (3.00;9.00)
RPE 90 min	3.50 [3.00;4.00] (3.00;5.00)	7.00 [6.00;7.00] (3.00;7.00)
RPE 105 min	4.00 [3.00;4.00] (3.00;6.00)	7.00 [7.00;8.00] (3.00;9.00)
RPE 120 min	4.00 [3.00;5.00] (3.00;6.00)	8.00 [8.00;8.50] (8.00;9.00)
THIRST PRE/REST	0.00 [0.00;0.00] (0.00;0.00)	0.00 [0.00;0.00] (0.00;0.00)
THIRST 15 min	2.00 [2.00;2.00] (2.00;3.00)	3.00 [3.00;4.00] (2.00;6.00)
THIRST 30 min	2.00 [2.00;2.50] (2.00;3.00)	3.00 [3.00;5.00] (2.00;6.00)
THIRST 45 min	3.00 [3.00;3.00] (2.00;3.00)	4.00 [4.00;6.00] (3.00;6.00)
THIRST 60 min	3.00 [3.00;3.00] (3.00;4.00)	6.00 [4.00;6.00] (3.00;8.00)
THIRST 75 min	4.00 [3.00;4.00] (3.00;4.00)	6.00 [6.00;7.00] (3.00;9.00)
THIRST 90 min	4.00 [4.00;5.00] (4.00;5.00)	7.00 [6.00;7.00] (4.00;7.00)
THIRST 105 min	5.00 [5.00;5.00] (4.00;5.00)	7.50[7.00;8.50] (5.00;9.00)
THIRST 120 min	5.00 [5.00;5.00] (5.00;6.00)	9.00 [8.50;9.00] (8.00;9.00)
HR PRE/REST	55.50 [53.00;63.50] (48.00;72.00)	61.00 [56.00;62.00] (48.00;71.00)
HR 15 min	127.00 [123.00;131.50] (104.00;143.00)	124.00 [122.00;128.00] (102.00;135.00)
HR 30 min	137.50 [127.50;142] (110.00;150.00)	130.00 [129.00;138.00] (110.00;146.00)
HR 45 min	143.00 [127.00;145.50] (110.00;154.00)	138.00 [131.00;143.00] (116.00;150.00)
HR 60 min	146.50 [132.50;148.00] (110.00;154.00)	141.00 [135.00;149.00] (121.00;156.00)
HR 75 min	149.50 [133.00;152.00] (111.00;156.00)	143.00 [135.00;151.00] (129.00;155.00)
HR 90 min	152.00 [135.50;157.50] (112.00;159.00)	146.00 [135.00;154.00] (129.00;164.00)
HR 105 min	156.00 [135.00;162.00] (114.00;165.00)	146.00 [138.00;152.00] (130.00;169.00)
HR 120 min	156.00 [135.00;164.00] (117.00;169.00)	154.00 [144.50;155.00] (135.00;156.00)

EXP: Experimental Group, CON: Control Group, Me: Median, IQR: Interquartile Range, min–max: range (minimum and maximum), T_c_: Core Temperature (°), RPE: Rating of Perceived Exertion (1–10), THIRST: Thirst Perception, HR: Heart Rate (BPM)

**Table 3:** Descriptive statistics of ΔMean and ΔPeak response variables (15–90 min).

Variable	EXP	CON
	Me [IQR] (min–max)	
ΔT_c_ 0–15 min	0.07 [0.05;0.08] (0.04;0.17)	0.07 [0.04;0.12] (−0.07;0.30)
ΔT_c_ 15–30 min	0.18 [0.09;0.27] (−0.30;1.92)	0.12 [0.02;0.38] (−0.07;0.70)
ΔT_c_ 30–45 min	0.16 [0.09;0.26] (−0.04;0.45)	0.10 [0.01;0.26] (−0.65;0.44)
ΔT_c_ T 45–60 min	0.19 [0.05;0.43] (−0.06;0.63)	0.05 [−0.03;0.20] (−0.05;0.40)
ΔT_c_ 60–75 min	0.05 [−0.02;0.27] (−0.29;0.95)	0.09 [−0.05;0.12] (−0.23;0.70)
ΔT_c_ 75–90 min	0.09 [−0.01;0.19] (−0.60;0.45)	0.01 [−0.13;0.13] (−0.43;0.23)
ΔT_c_ 90–105 min	0.12 [0.01;0.25] (−0.23;0.57)	0.02 [−0.03;0.06] (−0.10;0.22)
ΔT_c_ 105–120 min	0.05 [−0.02;0.10] (−0.21;0.15)	0.06 [0.04;0.17] (0.03;0.26)
Mean ΔT_c_	0.13 [0.09;0.17] (0.03;0.38)	0.08 [0.04;0.10] (−0.04;0.27)
Peak ΔT_c_	0.39 [0.28;0.58] (0.09;1.92)	0.36 [0.20;0.45] (0.12;0.70)
ΔRPE 0–15 min	1.00 [1.00;1.00] (1.00;1.00)	2.50 [2.00;3.00] (1.00;3.00)
ΔRPE 15–30 min	1.00 [1.00;1.00] (0.00;2.00)	1.00 [1.00;1.00] (0.00;1.00)
ΔRPE 30–45 min	1.00 [0.00;1.00] (0.00;2.00)	1.00 [1.00;1.00] (0.00;1.00)
ΔRPE 45–60 min	0.00 [0.00;1.00] (0.00;2.00)	1.00 [1.00;2.00] (0.00;3.00)
ΔRPE 60–75 min	0.00 [0.00;0.00] (0.00;1.00)	1.00 [0.00;1.00] (0.00;2.00)
ΔRPE 75–90 min	0.00 [0.00;0.50] (0.00;1.00)	1.00 [0.00;1.00] (0.00;2.00)
ΔRPE 90–105 min	0.00 [0.00;0.00] (0.00;1.00)	1.00 [0.50;1.00] (0.00;2.00)
ΔRPE 105–120 min	0.50 [0.00;1.00] (0.00;1.00)	1.00 [0.50;1.00] (0.00;1.00)
Mean ΔRPE	0.50 [0.46;0.63] (0.38;0.86)	1.00 [1.00;1.14] (0.43;1.80)
Peak ΔRPE	1.00 [1.00;1.00] (1.00;2.00)	2.50 [2.00;3.00] (1.00;3.00)
ΔTHIRST PRE/REST−15	2.00 [2.00;2.00] (2.00;3.00)	3.00 [3.00;4.00] (2.00;6.00)
ΔTHIRST 15–30 min	0.00 [0.00;0.00] (0.00;1.00)	0.00 [0.00;1.00] (0.00;1.00)
ΔTHIRST 30–45 min	1.00 [0.00;1.00] (0.00;1.00)	1.00 [1.00;1.00] (0.00;1.00)
ΔTHIRST 45–60 min	0.00 [0.00;0.00] (0.00;1.00)	0.50 [0.00;2.00] (0.00;2.00)
ΔTHIRST 60–75 min	1.00 [0.00;1.00] (0.00;1.00)	1.00 [0.00;1.00] (0.00;2.00)
ΔTHIRST 75–90 min	1.00 [0.00;1.00] (0.00;1.00)	0.00 [0.00;1.00] (0.00;2.00)
ΔTHIRST 90–105 min	1.00 [0.00;1.00] (0.00;1.00)	1.00 [1.00;2.00] (0.00;2.00)
ΔTHIRST 105–120 min	0.00 [0.00;0.50] (0.00;1.00)	0.50 [0.00;1.00] (0.00;1.00)
Mean ΔTHIRST	0.63 [0.63;0.71] (0.63;0.75)	1.13[1.00;1.14] (0.71;1.80)
Peak ΔTHIRST	2.00 [2.00;2.00] (2.00;3.00)	3.00 [3.00;4.00] (2.00;6.00)
ΔHR 0−15	70.50 [66.00;73.00] (50.00;86.00)	65.00 [60.00;69.00] (46.00;73.00)
ΔHR 15–30 min	7.00 [4.50;9.00] (2.00;17.00)	9.00 [6.00;10.00] (−4.00;16.00)
ΔHR 30–45 min	4.00 [1.00;6.00] (−2.00;7.00)	4.00 [2.00;6.00] (0.00;12.00)
ΔHR 45–60 min	2.00 [0.50;4.50] (0.00;8.00)	4.00 [3.00;5.00] (−1.00;13.00)
ΔHR 60–75 min	2.50 [1.00;4.50] (−2.00;7.00)	2.00 [0.00;3.00] (−5.00;8.00)
ΔHR 75–90 min	2.50 [2.00;6.00] (0.00;8.00)	3.00 [0.00;4.00] (−2.00;9.00)
ΔHR 90–105 min	3.00 [1.00;4.00] (0.00;6.00)	4.00 [−2.00;6.00] (−5.00;8.00)
ΔHR 105–120 min	3.00 [3.00;6.00] (0.00;8.00)	0.00 [−2.50;3.00] (−3.00;4.00)
Mean ΔHR	3.87 [2.43;5.14] (1.71;6.83)	3.50 [3.00;4.43] (1.71;6.83)
Peak ΔHR	7.00 [6.00;9.00] (5.00;17.00)	10.00 [8.00;11.00] (6.00;16.00)

EXP: Experimental Group, CON: Control Group, Me: Median, IQR: Interquartile Range, min–max: Range (Minimum and Maximum), T_c_: Core Temperature (°), RPE: Rating of Perceived Exertion (1–10), THIRST: ΔThirst Perception, HR: Heart Rate (BPM)

The group variable (EXP, CON) was significantly impacted by the exercise duration required to achieve a 3% body mass loss (χ^2^ = 10.57; *p* = 0.014; V_C_ = 0.55). The results indicated that participants in the EXP group most frequently reached exercise duration of 120 min (75.00% of participants), with the shortest duration being 90 min. In contrast, participants in the CON group most commonly achieved exercise duration of 105 min (57.14% of participants), with 28.57% completing the entire protocol, and the shortest duration recorded at 75 min.

### Core Temperature

A repeated-measures analysis of core temperature across seven time points (pre, 15–90 min) in the EXP group revealed a significant main effect of time (χ^2^(6, 16) = 73.31, *p* < 0.001, W = 0.76). Post hoc tests indicated higher temperature values at all post-baseline time points compared with pre (*p* < 0.001). Additional increases were observed between 15 and 30 min (*p* = 0.036), 15 and 45/60/75/90 min (all *p* < 0.001), 30 and 60/75/90 min (all *p* < 0.001), 45 and 60/75/90 min (all *p* < 0.001), as well as between 60 and 90 min (*p* = 0.005) and 75 and 90 min (*p* = 0.036). Comparisons between 30 and 45 min and between 60 and 75 min were non-significant (*p* = 0.168 and *p* = 0.463, respectively).

In the CON group, repeated-measures analysis also showed a significant effect of time, (χ^2^(6, 12) = 36.09, *p* < 0.001, W = 0.50). Core temperature did not differ between pre and 15 min (*p* = 0.14), but was significantly higher at 30, 45, 60, 75, and 90 min compared with pre (*p* ≤ 0.003). Further increases were observed between 15 and 45/60/75/90 min (all *p* ≤ 0.008), 30 and 60/75/90 min (*p* ≤ 0.02/0.001/< 0.001), and 45 and 75/90 min (*p* = 0.01/0.02). No significant changes were found between 30 and 45, 45 and 60, 60 and 75, 60 and 90, or 75 and 90 min (*p* = 0.283, 0.171, 0.233, 0.339, 0.811, respectively), suggesting a temperature rise until approximately 60–75 min, followed by a relative plateau.

Both Δ HR Mean and Δ HR Peak indicated no significant differences between the EXP and CON groups. For Δ Tc Mean, between-group differences in Δ Tc were non-significant (Δ Tc −0.03 (SE = 0.02; 95% CI –0.12; 0.15, t (27) = 0.22, *p* = 0.83; *d* = –0.29). For Δ Tc Peak, the difference was –0.04 (SE = 0.07; 95% CI –0.06; 0.08, t (27) = –1.60, *p* = 0.12; *d* = –0.11).

The EXP and CON groups differed in terms of the time to reach peak Tc (χ^2^ = 13.67; *p* = 0.03; VC = 0.63). The results indicate that participants in the EXP group most often achieved the highest Tc after 105 min and 120 min of exercise (for both 37.50%). On the other hand, participants in the CON group most often achieved peak Tc after 75 min (35.71% of participants) and 90 min and 105 min (for both 14.29%), and the shortest time to reach peak Tc was 60 min in both groups.

### Rating of Perceived Exertion (RPE)

A repeated-measures analysis of RPE across seven time points (pre, 15–90 min) in the EXP group demonstrated a significant main effect of time (χ^2^ (6, 16) = 89.15, *p* < 0.001, W = 0.93). Post hoc comparisons indicated progressive increases in RPE values across all time points (*p* < 0.001), with significant differences between 75 and 90 min (*p* = 0.04) except for 60 vs. 75 min (*p* = 0.09).

Similarly, in the CON group, RPE analysis revealed a significant effect of time (χ^2^ (6, 12) = 69.59, *p* < 0.001, W = 0.97). Post hoc tests showed consistent increases across all time points (*p* < 0.001).

The CON group exhibited higher mean differences than the EXP group. For Δ RPE Mean, the between-group difference was +0.23 (SE = 0.05; 95% CI 0.13; 0.34, t (27) = 4.62, *p* < 0.001, *d* = 0.89), while for Δ RPE Peak, the between-group difference was +0.53 (SE = 0.10; 95% CI 0.32; 0.74, t (27) = 5.12, *p* < 0.001, *d* = 1.02).

The two groups (EXP and CON) did not differ in time to reach peak RPE (χ^2^ = 8.50; *p* = 0.13; VC = 0.48). Peak RPE was mainly reached in a time window of 90 min–120 min.

### Thirst Perception

A repeated-measures analysis of thirst perception across seven time points (pre, 15–90 min) in the EXP group revealed a significant effect of time (χ^2^(6, 16) = 90.04, *p* < 0.001, W = 0.94). Post hoc tests showed increased thirst ratings across all time points (*p* < 0.001), with significant differences between 15 and 30 min (*p* = 0.04) and between 45 and 60 min (*p* = 0.19).

In the CON group, a significant effect of time was also detected (χ^2^(6, 16) = 69.40, *p* < 0.001, W = 0.96). Post hoc analysis revealed higher thirst ratings across all time points (*p* < 0.001), with additional differences at 75 and 90 min (*p* = 0.001), while comparisons between 15 and 30 min were not significant (*p* = 0.06).

The CON group exhibited higher mean differences than the EXP group. For Δ Thirst Mean, the between-group difference was +0.23 (SE = 0.04; 95% CI 0.14; 0.32, t (27) = 5.23, *p* < 0.001; *d* = 1.11). For Δ Thirst Peak, the between-group difference was +0.65 (SE = 0.17; 95% CI 0.30; 1.01, t (27) = 3.79, *p* < 0.001; *d* = 0.74).

Both groups (EXP and CON) did not differ in time to peak Thirst (χ^2^ = 4.17; *p* = 0.24; VC = 0.34). Peak Thirst was mainly reached within the 90–120-min time window.

### Heart Rate (HR)

A repeated-measures analysis of the HR across seven time points (pre, 15–90 min) in the EXP group demonstrated a significant main effect of time (χ^2^ (6, 16) = 93.69, *p* < 0.001, W = 0.97). Post hoc tests showed consistent increases across all time points (*p* < 0.001).

Similarly, in the CON group, HR analysis revealed a significant effect of time (χ^2^ (6, 12) = 63.11, *p* < 0.001, W = 0.95). Post hoc comparisons indicated progressive increases in RPE values across all time points (*p* < 0.001), with significant differences between 75 and 90 min (*p* = 0.001).

Both Δ HR Mean and Δ HR Peak indicated no significant differences between the EXP and CON groups. For Δ HR Mean, the difference between groups was –0.41 (SE = 0.29; 95% CI – 1.01; 0.19, t (25) = –1.40, *p* = 0.18, *d* = –0.55). For the Δ RPE Peak, the difference between groups was –0.11 (SE = 0.57; 95% CI –1.07; 1.29, t (25) = 0.19, *p* = 0.85, *d* = 0.08).

Both groups differed in terms of the time to reach peak HR (χ^2^ = 10.77; *p* = 0.029; VC = 0.57). The results indicate that participants in the EXP group most often reached peak HR within 120 min of exercise (68.75%), with the earliest point of reaching peak HR being 90 min. In contrast, participants in the CON group most often reached peak HR after 105 min (46.15% of participants), with the earliest time point of reaching peak HR being after 45 min.

Spearman’s rank correlation coefficients revealed significant positive associations between Δ RPE and BF%, USG, and Δ Thirst (rcorr = 0.39, 0.54, and 0.84, respectively; all *p* < 0.05), as well as negative associations with VO_2__max_ and Δ Tc (rcorr = −0.62, and −0.38, respectively). Δ Thirst was significantly and positively correlated with USG (rcorr = 0.62; *p* < 0.05) and negatively correlated with VO_2__max_ (rcorr = −0.45; *p* < 0.05). A significant positive relationship was also observed between Δ Tc and the average sweat rate (rcorr = 0.57; *p* < 0.05), and a significant negative correlation between VO_2__max_ and BF% (rcorr = −0.44; *p* < 0.05).

In the EXP group, Spearman’s rank correlations indicated a significant negative association with Δ RPE (rcorr = −0.61; *p* < 0.05). Δ Tc correlated positively with Δ Thirst and the average sweat rate (rcorr = 0.53 and 0.73, respectively; all *p* < 0.05). Δ RPE was also positively correlated with BF% (rcorr = 0.64; *p* < 0.05). Δ HR correlated positively with Δ Tc and Δ RPE (rcorr = 0.51, and −0.56, respectively, all *p* < 0.05).

In the CON group, Spearman’s rank correlations revealed significant associations between VO_2__max_ and FM%, Δ RPE, and Δ Thirst (rcorr = −0.58, −0.67, and −0.65, respectively; all *p* < 0.05), as well as a significant positive association between Δ Thirst and Δ RPE (rcorr = 0.96; *p* < 0.05).

An ANCOVA with the RPE as the dependent variable revealed a statistically significant overall model (F (3,26) = 13.76, *p* < 0.001). The main effect of Tc was not statistically significant (F (1,26) = 0.06, *p* = 0.81, partial η^2^ = 0.002). Hydration status emerged as a significant predictor (F (1,26) = 18.18, *p* < 0.001, partial η^2^ = 0.41), while VO_2__max_ showed a not significant effect (F (1,26) = 3.93, *p* = 0.058, partial η^2^ = 0.13).

The ANCOVA for thirst was significant overall (F (3,25) = 13.91, *p* < 0.001). Pre-exercise USG did not show a significant main effect (F (1,25) = 0.93, *p* = 0.35, partial η^2^=0.04). Hydration status remained a significant predictor (F (1,25) = 9.41, *p* = 0.005, partial η^2^=0.27), while VO_2__max_ did not reach significance (F (1,25) = 4.20, *p* = 0.051, partial η^2^ = 0.14).

Further analysis of covariance (ANCOVA), with perception of thirst as the dependent variable, revealed a statistically significant overall model (F (3,28) = 12.84, *p* < 0.001). The main effect of the body fat percentage was not statistically significant (F (1,28) = 0.002, *p* = 0.97, partial η^2^ < 0.001). However, hydration status remained a significant predictor (F (1,28) = 25.91, *p* < 0.001, partial η^2^ = 0.50), while VO_2__max_ again failed to reach statistical significance (F (1,28) = 0.52, *p* = 0.48, partial η^2^ = 0.02).

## Discussion

This study examined how core temperature (Tc), ratings of perceived exertion (RPEs), and thirst evolved during 120 min of moderate cycling under different baseline hydration states. Across both groups, Tc rose from rest and then tended to stabilize after ~60–75 min, whereas RPEs and thirst increased throughout the trial, indicating a temporal dissociation between thermal kinetics and perceptual drift under thermoneutral laboratory conditions. When potential confounders were considered, hydration status, not Tc per se, emerged as the dominant predictor of both RPEs and thirst. Moreover, aerobic fitness (VO_2__max_) showed, at most, a trend-level association (*p* ≈ 0.05) that did not meet conventional significance. Baseline hydration also appeared to influence tolerance to moderate-intensity exercise. Participants who began trials less well hydrated were disproportionately likely to terminate the bout before completing 120 min of exercise. Correlational analyses supported a coupling between subjective states (RPE and thirst; very strong) and between these states and physiological strain (HR and Tc; moderate). Together, these findings suggest that starting exercise euhydrated is associated with lower perceived effort and thirst during prolonged, thermoneutral exercise, even when Tc differences are small. Notably, the strong within-trial coupling between RPE and thirst should be interpreted as correlational; the design does not establish directionality, and both perceptions may be co-driven by shared physiological signals related to fluid balance and cardiovascular/thermal strain.

### Results in Relation to Current Evidence

The present findings are in accordance with a substantial body of evidence indicating that hydration status exerts an independent and practically meaningful influence on ratings of perceived exertion during prolonged exercise. The applied importance of baseline hydration is supported by field studies in soccer showing that athletes often begin sessions and competitions hypohydrated and may underconsume fluids during play (Klimesova et al., 2022; Fernández-Álvarez et al., 2022). Meta-analytic estimates suggest a dose-response relation between exercise-induced dehydration (EID) and RPE (≈ +0.21 Borg units per 1% body-mass loss), with more pronounced effects typically observed beyond ~3% loss. Nevertheless, smaller decrements can still shift exertional perception, particularly as task duration accumulates. In this study, primary analyses were limited to 90 min to minimize adverse effects of higher EID, yet between-condition differences in RPE were already apparent, consistently with the hypothesis that even mild hypohydration can “prime” perceptual responses (Deshayes et al., 2022).

The temporal profile of Tc, a rapid early rise followed by attenuation toward a quasi-steady state aligns with established thermoregulatory models for moderate, fixed-intensity exercise under thermoneutral conditions. Notably, RPE continued to increase after Tc had begun to plateau. This dissociation is compatible with prior work indicating that integrated cardiovascular and metabolic strain, together with central processing of thermal and non-thermal afferents, progressively biases perceived effort over time, even when Tc variability is constrained. Our results converge with extensive literature in suggesting that hydration status is a salient determinant of exertional perception independent of Tc, and the kinetics of RPE during steady work is only partially explained by Tc, with additional contributions from cardiovascular load, fluid-electrolyte balance, and central integrative mechanisms (Périard et al., 2021).

Under thermoneutral, compensable conditions, the early rise in core temperature reflects transient heat storage while thermoeffector responses such as cutaneous vasodilation and sweating increase gradually. As evaporative and dry heat loss increasingly match metabolic heat production, net heat storage approaches zero and Tc can reach a quasi-steady state, producing an apparent plateau after approximately 60 to 75 min even though exercise continues (Sawka et al., 1993). Importantly, a Tc plateau does not imply that internal strain is no longer accumulating. Thirst can continue to rise because sweat loss progressively reduces total body water and plasma volume while concentrating body fluids, which increases osmotic and volume related drive to drink. Experimental exercise heat models show that hypohydration before and during exercise increases thirst and fluid regulatory hormonal responses relative to euhydration (Maresh et al., 2004). In parallel, RPE may continue to increase over time because dehydration and prolonged exercise promote cardiovascular and metabolic drift at a fixed external workload, including reductions in stroke volume and plasma volume with compensatory tachycardia and greater substrate strain, which heighten perceived effort even when Tc changes are small (Montain and Coyle, 1992; Logan-Sprenger et al., 2012). This interpretation is consistent with meta-analytic evidence demonstrating a dose response increase in perceived exertion as dehydration accrues, with effects becoming more evident at higher levels of body mass loss (Deshayes et al., 2022).

### Mechanism Consideration

Hydration status likely influenced RPE through multiple pathways, including reduced plasma volume, a higher heart rate for a given workload, and greater perceived strain mechanisms long implicated in heat and dehydration related performance impairment (Périard et al., 2021). Our moderate positive RPE-HR and RPE-Tc correlations and the strong RPE-thirst coupling support this integrated view of cardiovascular and thermoregulatory strain converging on perception. Moreover, hyperosmolality is a principal driver of thirst. Body-fluid signals (e.g., osmolality, AVP) integrate with thermal inputs so that a higher Tc can amplify thirst drive at a given hydration deficit (Antunes-Rodrigues et al., 2004). The present data, where hydration status predicted thirst even after accounting for USG, fit with models in which dynamic, brain-wide fluid-balance networks respond to both osmotic and thermal cues rather than any single surrogate marker.

Tc did not independently predict RPE in the adjusted models. This is also coherent with contemporary frameworks. When Tc variability is constrained (thermoneutral lab, fixed workload, partial Tc plateau), perceptual drift may be driven predominantly by cardiovascular strain, substrate use, and central fatigue processes, with hydration modulating these pathways upstream of Tc. Prior research shows that heat stress and dehydration reduce central drive and increase perceived effort at a given workload, even before Tc nears critical thresholds (Nybo et al., 2015). Recent data further indicate that manipulating systemic buffering capacity via sodium bicarbonate supplementation can enhance lactate efflux and improve anaerobic and cognitive performance in elite combat-sport athletes, underscoring potential links between peripheral metabolic milieu and subjective responses to exertion (Chycki et al., 2021; Skalski et al., 2024, 2025a, 2025b, 2025c,2025d; Prończuk et al. 2024, 2025).

Interpreting our findings in light of physiological sequence, available evidence supports thermal and fluid-electrolyte perturbations as upstream drivers of both perceived exertion and thirst, rather than these perceptions cause increases in Tc. Hyperthermia can directly constrain endurance capacity, with high internal temperature shown to precipitate fatigue during prolonged exercise in uncompensable heat (González-Alonso et al., 1999). Complementarily, in fixedpower cycling, the rate of increase in RPE predicts time to exhaustion and rises rectal temperature across environmental conditions, consistently with RPE tracking accumulating physiological strain (Crewe et al., 2008). Thirst is similarly justified in osmotic and volume regulation. Because effective extracellular tonicity is governed largely by sodium salts, progressive hypotonic sweat losses tend to concentrate plasma electrolytes, particularly Na⁺ with its attendant anion Cl⁻, thereby increasing plasma osmolality and activating osmosensitive structures of the lamina terminalis, including the OVLT and SFO, to promote arginine vasopressin release and drinking behavior (Stachenfeld, 2014; Wang et al., 2023). In laboratory models, rapid elevations in plasma sodium and osmolality induced by hypertonic NaCl elicit robust increases in thirst and vasopressin, underscoring a causal role of NaCl driven osmotic stimulation in human thirst regulation (Stachenfeld, 2014).

Moreover, experimentally elevating core temperature augments osmotically induced thirst and vasopressin responses, indicating that thermal strain can amplify the thirst drive at a given osmotic stimulus (Takamata et al., 1995). Conversely, when NaCl is lost disproportionately in sweat, the rise in body fluid osmolality may be blunted and thirst drive attenuated despite substantial sweating, as shown in children with cystic fibrosis exercising in the heat, where high sweat NaCl losses were associated with reduced thirst guided drinking and greater dehydration (Bar-Or et al., 1992), and where increasing beverage NaCl content increased voluntary fluid intake and mitigated progressive dehydration alongside declines in serum sodium and chloride (Kriemler et al., 1999). At the same time, in self-paced paradigms, RPE can function as a regulatory control signal, such that maintaining a fixed RPE elicits anticipatory reductions in power output that limit heat storage (Tucker et al., 2004, 2006). Accordingly, our study design likely emphasizes the feedforward pathway, with Tc and hydration related perturbations shaping perceptions, whereas reciprocal feedback of perception on physiology is expressed primarily as earlier task disengagement in less hydrated participants.

It is worth noting that significant Time × VO_2__max_ interactions for RPE and thirst indicate that higher fitness blunted growth in perceived strain and thirst over time. Enhanced heat dissipation, plasma volume expansion, and improved cardiovascular efficiency in fitter individuals plausibly underlie this moderation effect, in line with integrative reviews on performance in the heat (Périard et al., 2021). VO_2__max_, is generally associated with more efficient thermoregulation through adaptations such as plasma volume expansion and improved cutaneous vasodilation and sweating responsiveness, which together reduce cardiovascular and thermal strain during exercise, particularly in hot environments (Périard et al., 2021). However, this relationship is not always evident when exercise is performed at a fixed relative intensity and under compensable conditions, because trained individuals often sustain higher absolute metabolic heat production, and group differences in Tc may be attenuated or even reversed under matched relative workloads (Mora-Rodriguez et al., 2010, 2012). In the present study, the individualized moderate workload and thermoneutral laboratory setting likely constrained Tc variability and promoted a quasi-steady state thermal profile, which may explain why a clear VO_2__max_ related advantage in Tc responses was not observed after accounting for hydration status and other covariates (Périard et al., 2021).

### Practical Implications

For coaches, nutritionists, and practitioners, two points stand out. First off all, arriving euhydrated and following a structured drinking plan reduces perceived effort and thirst during prolonged sessions, even in thermoneutral labs, making adherence more likely as duration increases. Secondly, because perception was tightly coupled to the HR and thirst, simple field-ready measures (HR monitoring, thirst scales) can serve as early warning signs of inadequate hydration before Tc diverges meaningfully among athletes. These recommendations complement existing guidelines on pre-, during-, and post-exercise hydration strategies.

### Future Directions

Future research should (i) incorporate direct assessments of plasma or serum osmolality to quantify osmotic drive alongside urine specific gravity and body-mass change; (ii) interrogate hotter and cooler environments and implement variable workloads to broaden the characterization of Tc dynamics; (iii) include women and participants with heterogeneous training status; and (iv) experimentally manipulate drinking strategies and perceptual cues to more rigorously test causality within the Tc-hydration-perception relationship; and (v) include criterion core-temperature measures (e.g., gastrointestinal telemetric capsules) in at least a subsample to quantify agreement and potential bias of heat-flux sensors across steady-state and non-steady-state conditions.

### Limitations

Our findings should be interpreted in light of several constraints. Participants were physically active adult males, thus generalization to women or to elite and youth populations requires caution. Exercise occurred under thermoneutral conditions. Hotter or colder environments may alter the Tc-perception coupling and magnify hydration effects. Primary analyses were cut short at 90 min to mitigate attrition bias from dropouts occurring around 3% body-mass loss, although statistically justified. This decision limits inference at more severe levels of exercise-induced dehydration, where perceptual effects may be larger. Finally, although the core-temperature method is validated for steady cycling, any residual bias would attenuate Tc effects, potentially pushing variance toward cardiovascular and hydration predictors in adjusted models. In addition, participants represented a moderately broad adult age distribution (mean 34.67 ± 6.12 years). Although age did not differ meaningfully between groups, it constitutes an important physiological moderator, given that thermoregulatory effectiveness and cardiovascular function can vary across adulthood, including changes in sweating responsiveness, cutaneous perfusion, and overall heat dissipation capacity. Consequently, modest age-related heterogeneity may have contributed to inter-individual variability in Tc trajectories, perceptual responses, and exercise tolerance, and the present findings should therefore be generalized with caution to substantially younger or older populations.

Moreover, we used a non-invasive heat-flux-based core-temperature sensor. Heat-flux systems estimate Tc via biophysical modeling and algorithmic correction rather than directly measuring internal temperature; therefore, absolute values can differ from criterion methods such as rectal, esophageal, or gastrointestinal telemetric measurements. Potential sources of error include local skin perfusion changes, sweating, sensor placement, and movement artifacts. Additionally, the magnitude/direction of bias may vary across environments and when core temperature is changing rapidly. A validation study has reported acceptable agreement versus gastrointestinal references during cycling and controlled heat exposure contexts similar to our steady, moderate-intensity protocols (Jolicoeur Desroches et al., 2023). That said, some studies suggest underestimation at high, rapidly changing intensities (e.g., 5-km time trials in heat), conditions distinct from the present study (Goods et al., 2023; Verdel et al., 2021). These data support the suitability of our approach for steady, moderate-intensity cycling, while acknowledging scenario-specific limitations. Importantly, any non-differential measurement error in Tc would be expected to attenuate associations with perceptual outcomes, which may partly contribute to the limited independent contribution of Tc observed in the adjusted models.

Baseline differences in aerobic fitness (e.g., VO_2__max_) were present between groups and despite covariate adjustment and at most a trend-level association (*p* ≈ 0.05), residual confounding cannot be fully excluded, as such disparities may have influenced perceptual and physiological trajectories as well as task tolerance. To mitigate this risk, all exercise workloads were individualized and prescribed on a relative basis (e.g., as a percentage of each participant’s maximal aerobic capacity) anchored to the screening/qualification assessment, thereby minimizing the potential disproportionate impact of higher baseline fitness.

## Conclusions

In prolonged, moderate-intensity cycling, baseline hydration status exerted a robust and independent influence on exertional and thirst perceptions, whereas core temperature (Tc) contributed little to RPE after accounting for hydration and aerobic fitness. The temporal dissociation of RPE rising despite a Tc plateau underscores the primacy of fluid-balance and cardiovascular strain pathways over Tc per se in shaping perceived effort under thermoneutral, steady-load conditions. Practically, ensuring euhydration before exercise and adopting structured fluid strategies during exercise are likely to attenuate perceived effort and thirst, thereby supporting adherence and performance. Simple, field-ready markers, thirst ratings and heart rate monitoring offer optimal tools to identify emerging strain, with particular utility among individuals with lower VO_2__max_. Collectively, these findings refine the mechanistic account of the Tc-hydration-perception relationship and provide clear, implementable guidance for training and athlete support.

From an applied perspective, the results support the practical importance of starting exercise euhydrated and using structured hydration strategies, particularly for longer sessions where perceived strain progressively accumulates. In field settings, simple, low-burden monitoring tools such as heart-rate tracking and brief thirst ratings may help identify emerging hydration-related strain before meaningful divergence in Tc is apparent, with particular relevance for individuals with lower aerobic capacity or poorer baseline hydration.

## References

[ref1] Antunes-Rodrigues, J., de Castro, M., Elias, L. L. K., Valença, M. M., & McCann, S. M. (2004). Neuroendocrine control of body fluid metabolism. *Physiological Reviews*, 84(1), 169–208. 10.1152/physrev.00017.2003 14715914

[ref2] Armstrong, L. E., Maresh, C. M., Castellani, J. W., Bergeron, M. F., Kenefick, R. W., LaGasse, K. E., & Riebe, D. (1994). Urinary indices of hydration status. *International Journal of Sport Nutrition*, 4(3), 265–279. 10.1123/ijsn.4.3.265 7987361

[ref3] Armstrong, L. E., & Kavouras, S. A. (2019). Thirst and drinking paradigms: Evolution from single factor effects to brainwide dynamic networks. *Nutrients*, 11(12), 2864. 10.3390/nu11122864 31766680 PMC6950074

[ref4] Bar-Or, O., Blimkie, C. J., Hay, J. A., MacDougall, J. D., Ward, D. S., & Wilson, W. M. (1992). Voluntary dehydration and heat intolerance in cystic fibrosis. *Lancet*, 339(8795), 696–699. 10.1016/0140-6736(92)90597-V 1347582

[ref5] Bichet, D. G. (2018). Vasopressin and the regulation of thirst. *Annals of Nutrition and Metabolism*, 72(Suppl 2), 3–7. 10.1159/000488233 29925072

[ref6] Borg, G. (1998). Borg’s perceived exertion and pain scales. Human Kinetics.

[ref7] Cao, Y., Lei, T.-H., Wang, F., Yang, B., & Mündel, T. (2022). Head, face and neck cooling as per-cooling (cooling during exercise) modalities to improve exercise performance in the heat: A narrative review and practical applications. *Sports Medicine-Open*, 8(1), 16. 10.1186/s40798-022-00411-4 35092517 PMC8800980

[ref8] Casa, D. J., Armstrong, L. E., Hillman, S. K., Montain, S. J., Reiff, R. V., Rich, B. S. E., Roberts, W. O., & Stone, J. A. (2000). National Athletic Trainers’ Association position statement: Fluid replacement for athletes. *Journal of Athletic Training*, 35(2), 212–224. 16558633 PMC1323420

[ref9] Cheuvront, S. N., Kenefick, R. W., Montain, S. J., & Sawka, M. N. (2010). Mechanisms of aerobic performance impairment with heat stress and dehydration. *Journal of Applied Physiology*, 109(6), 1989–1995. 10.1152/japplphysiol.00367.2010 20689090

[ref10] Chycki, J., Zając, T., Maszczyk, A., & Kurylas, A. (2017). The effect of mineral-based alkaline water on hydration status and the metabolic response to short-term anaerobic exercise. *Biology of Sport*, 34(3), 255–261. 10.5114/biolsport.2017.66003 29158619 PMC5676322

[ref11] Chycki, J., Kurylas, A., Maszczyk, A., Gołaś, A., & Zając, A. (2018). Alkaline water improves exercise-induced metabolic acidosis and enhances anaerobic exercise performance in combat sport athletes. *PLoS One*, 13(11), e0205708. 10.1371/journal.pone.0205708 30452459 PMC6242303

[ref12] Chycki, J., Zając, A., & Toborek, M. (2021). Bicarbonate supplementation via lactate efflux improves anaerobic and cognitive performance in elite combat sport athletes. *Biology of Sport*, 38(4), 545–553. 10.5114/biolsport.2020.96320 34937963 PMC8670805

[ref13] Chiron, F., Thomas, C., Bardin, J., Mullie, F., Bennett, S., Chéradame, J., Caliz, L., Hanon, C. & Tiollier, E. (2024). Influence of Ingestion of Bicarbonate-Rich Water Combined with an Alkalizing or Acidizing Diet on Acid-Base Balance and Anaerobic Performance. *Journal of Human Kinetics*, 93, 105–117. 10.5114/jhk/182986 39132426 PMC11307191

[ref14] Cohen, J. (1988). *Statistical power analysis for the behavioral sciences* (2nd ed.). Lawrence Erlbaum Associates.

[ref15] Crewe, H., Tucker, R., & Noakes, T. D. (2008). The rate of increase in rating of perceived exertion predicts the duration of exercise to fatigue at a fixed power output in different environmental conditions. *European Journal of Applied Physiology*, 103(5), 569–577. 10.1007/s00421-008-0741-7 18461352

[ref16] Daanen, H. A. M., Kohlen, V., & Teunissen, L. P. J. (2023). Heat flux systems for body core temperature assessment during exercise. *Journal of Thermal Biology*, 112, 103480. 10.1016/j.jtherbio.2023.103480 36796923

[ref17] Deshayes, T. A., Pancrate, T., & Goulet, E. D. B. (2022). Impact of dehydration on perceived exertion during endurance exercise: A systematic review with meta-analysis. *Journal of Exercise Science & Fitness*, 20(3), 224–235. 10.1016/j.jesf.2022.03.006 35601980 PMC9093000

[ref18] Jolicoeur Desroches, A., Naulleau, C., Deshayes, T. A., Pancrate, T., & Goulet, E. D. B. (2023). CORE™ wearable sensor: Comparison against gastrointestinal temperature during cold water ingestion and a 5 km running time-trial. *Journal of Thermal Biology*, 115, 103622 37352596 10.1016/j.jtherbio.2023.103622

[ref19] Engell, D. B., Maller, O., Sawka, M. N., Francesconi, R. N., Drolet, L., & Young, A. J. (1987). Thirst and fluid intake following graded hypohydration levels in humans. *Physiology & Behavior*, 40(2), 229–236. 10.1016/0031-9384(87)90212-5 3306730

[ref20] EFSA Panel on Dietetic Products, Nutrition and Allergies (NDA). (2010). Scientific opinion on dietary reference values for water. *EFSA Journal*, 8(3), 1459. 10.2903/j.efsa.2010.1459

[ref21] Fernández-Álvarez, M. D. M., Cachero-Rodríguez, J., Leirós-Díaz, C., Carrasco-Santos, S., & Martín-Payo, R. (2022). Evaluation of water intake in Spanish adolescent soccer players during a competition. *Journal of Human Kinetics*, 83, 59–66. 10.2478/hukin-2022-0051 36157942 PMC9465763

[ref22] Flouris, A. D., & Schlader, Z. J. (2015). Human behavioral thermoregulation during exercise in the heat. *Scandinavian Journal of Medicine & Science in Sports*, 25(S1), 52–64. 10.1111/sms.12349 25943656

[ref23] Goods, P. S. R., Maloney, P., Miller, J., Jennings, D., Fahey-Gilmour, J., Peeling, P., & Galna, B. (2023). Concurrent validity of the CORE wearable sensor with BodyCap temperature pill to assess core body temperature during an elite women's field hockey heat training camp. *European Journal of Sport Science*, 23(8), 1509–1517. 36939844 10.1080/17461391.2023.2193953

[ref24] González-Alonso, J., Teller, C., Andersen, S. L., Jensen, F. B., Hyldig, T., & Nielsen, B. (1999). Influence of body temperature on the development of fatigue during prolonged exercise in the heat. *Journal of Applied Physiology*, 86(3), 1032–1039. 10.1152/jappl.1999.86.3.1032 10066720

[ref25] Greenleaf, J. E. (1992). Problem: Thirst, drinking behavior, and involuntary dehydration. *Medicine & Science in Sports & Exercise*, 24(6), 645–656. 10.1249/00005768-199206000-00007 1602937

[ref26] Hew-Butler, T., Loi, V., Pani, A., & Rosner, M. H. (2017). Exercise-associated hyponatremia: 2017 update. *Frontiers in Medicine*, 4, 21. 10.3389/fmed.2017.00021 28316971 PMC5334560

[ref27] Januário, W. M., Lessa, N. F., Schittine, A. J. O., Prata, E. R. B. A., Marins, J. C. B., Natali, A. J., Wanner, S. P., & Prímola-Gomes, T. N. (2024). Validity and reproducibility of the CALERA research sensor to estimate core temperature at different intensities of a cycling exercise in the heat. *Journal of Thermal Biology*, 123, 103907. 10.1016/j.jtherbio.2024.103907 38950497

[ref28] Kenefick, R. W., Hazzard, M. P., Mahood, N. V., & Castellani, J. W. (2004). Thirst sensations and AVP responses at rest and during exercise-cold exposure. *Medicine & Science in Sports & Exercise*, 36(9), 1528–1534. 10.1249/01.MSS.0000139901.63911.75 15354034

[ref29] Klimesova, I., Krejci, J., Botek, M., McKune, A. J., Jakubec, A., Neuls, F., & Valenta, M. (2022). Prevalence of dehydration and the relationship with fluid intake and self-assessment of hydration status in Czech First League soccer players. *Journal of Human Kinetics*, 82, 101–110. 10.2478/hukin-2022-0035 36157002 PMC9465733

[ref30] Kriemler, S., Wilk, B., Schurer, W., Wilson, W. M., & Bar-Or, O. (1999). Preventing dehydration in children with cystic fibrosis who exercise in the heat. *Medicine & Science in Sports & Exercise*, 31(6), 774–779. 10.1097/00005768-199906000-00003 10378902

[ref31] Logan-Sprenger, H. M., Heigenhauser, G. J. F., Killian, K. J., & Spriet, L. L. (2012). Effects of dehydration during cycling on skeletal muscle metabolism in females. *Medicine & Science in Sports & Exercise*, 44(10), 1949–1957. 10.1249/MSS.0b013e31825abc7c 22543739

[ref32] Magee, P. J., Gallagher, A. M., & McCormack, J. M. (2017). High prevalence of dehydration and inadequate nutritional knowledge among university and club level athletes. *International Journal of Sport Nutrition and Exercise Metabolism*, 27(2), 158–168. 10.1123/ijsnem.2016-0053 27710146

[ref33] Maresh, C. M., Gabaree-Boulant, C. L., Armstrong, L. E., Judelson, D. A., Hoffman, J. R., Castellani, J. W., Kenefick, R. W., Bergeron, M. F., & Casa, D. J. (2004). Effect of hydration status on thirst, drinking, and related hormonal responses during low-intensity exercise in the heat. *Journal of Applied Physiology*, 97(1), 39–44. 10.1152/japplphysiol.00956.2003 14990557

[ref34] McDermott, B. P., Anderson, S. A., Armstrong, L. E., Casa, D. J., Cheuvront, S. N., Cooper, L., Kenney, W. L., O’Connor, F. G., & Roberts, W. O. (2017). National Athletic Trainers’ Association position statement: Fluid replacement for the physically active. *Journal of Athletic Training*, 52(9), 877–895. 10.4085/1062-6050-52.9.02 28985128 PMC5634236

[ref35] Montain, S. J., & Coyle, E. F. (1992). Influence of graded dehydration on hyperthermia and cardiovascular drift during exercise. *Journal of Applied Physiology*, 73(4), 1340–1350. 10.1152/jappl.1992.73.4.1340 1447078

[ref36] Mora-Rodriguez, R., Del Coso, J., Hamouti, N., Estevez, E., & Ortega, J. F. (2010). Aerobically trained individuals have greater increases in rectal temperature than untrained ones during exercise in the heat at similar relative intensities. *European Journal of Applied Physiology*, 109(5), 973–981. 10.1007/s00421-010-1436-4 20349316

[ref37] Mora-Rodriguez, R. (2012). Influence of aerobic fitness on thermoregulation during exercise in the heat. *Exercise and Sport Sciences Reviews*, 40(2), 79–87. 10.1097/JES.0b013e318246ee56 22205388

[ref38] Nybo, L., & González-Alonso, J. (2015). Critical core temperature: A hypothesis too simplistic to explain hyperthermia-induced fatigue. *Scandinavian Journal of Medicine & Science in Sports*, 25(S1), 4–5. 10.1111/sms.12444 25943652

[ref39] Périard, J. D., Eijsvogels, T. M. H., & Daanen, H. A. M. (2021). Exercise under heat stress: Thermoregulation, hydration, performance implications, and mitigation strategies. *Physiological Reviews*, 101(4), 1873–1979. 10.1152/physrev.00038.2020 33829868

[ref40] Pilch, W., Żychowska, M., Piotrowska, A., Czerwińska-Ledwig, O., Mikuľáková, W., & Sadowska-Krępa, E. (2022). Effects of elevated body temperature on selected physiological indices and thermal stress in athletes and non-athletes. *Journal of Human Kinetics*, 84, 112–123. 10.2478/hukin-2021-0131 36457467 PMC9679193

[ref41] Prończuk, M., Motowidło, J., Lulińska, E., Skalski, D. T., Markowski, J., Pilch, J., Kostrzewa, M., Terbalyan, A., Skotniczny, K., & Maszczyk, A. (2024). The effect of 6-week EEG-biofeedback training in normobaric hypoxia and normoxia conditions on reaction time in elite judo athletes. *Baltic Journal of Health and Physical Activity*, 16(3), 3. 10.29359/BJHPA.16.3.03

[ref42] Prończuk, M., Skalski, D., Łosińska, K., Maszczyk, A., & Gołaś, A. (2025). Neuromechanical adaptations to EMG-guided SSC training in elite badminton players: a predictive multivariate approach. *Frontiers in Sports and Active Living*, 7, 1634656. 10.3389/fspor.2025.1634656 41018810 PMC12460308

[ref43] Salata, R. A., Verbalis, J. G., & Robinson, A. G. (1987). Cold water stimulation of oropharyngeal receptors in man inhibits release of vasopressin. *Journal of Clinical Endocrinology & Metabolism*, 65(3), 561–567. 10.1210/jcem-65-3-561 3624414

[ref44] Sawka, M. N., & Pandolf, K. B. (1990). Effects of body water loss on exercise performance and physiological functions. In C. V. Gisolfi & D. R. Lamb (Eds.), *Perspectives in exercise science and sports medicine: Vol. 3. Fluid homeostasis during exercise* (pp. 1–38). Benchmark Press.

[ref45] Sawka, M. N., Wenger, C. B., Young, A. J., & Pandolf, K. B. (1993). Physiological responses to exercise in the heat. In B. M. Marriott (Ed.), *Nutritional needs in hot environments: Applications for military personnel in field operations* (pp. 55–74). National Academies Press. 10.17226/2094 25144014

[ref46] Sawka, M. N., Burke, L. M., Eichner, E. R., Maughan, R. J., Montain, S. J., & Stachenfeld, N. S. (2007). American College of Sports Medicine position stand: Exercise and fluid replacement. *Medicine & Science in Sports & Exercise*, 39(2), 377–390. 10.1249/mss.0b013e31802ca597 17277604

[ref47] Skalski, D., Kostrzewa, M., Prończuk, M., Markowski, J., Pilch, J., Żak, M., & Maszczyk, A. (2024). The effect of EEG biofeedback training frequency and environmental conditions on simple and complex reaction times. *Bioengineering*, 11(12), 1208. 10.3390/bioengineering11121208 39768026 PMC11673860

[ref48] Skalski, D., Prończuk, M., Stastny, P., Łosińska, K., Drozd, M., Toborek, M., Aschenbrenner, P., & Maszczyk, A. (2025a). Evaluation of the Impact of External Conditions on Arm Positioning During Punches in MMA Fighters: A Comparative Analysis of 2D and 3D Methods. *Sensors (Basel, Switzerland)*, 25(11), 3270. 10.3390/s25113270 40968810 PMC12157019

[ref49] Skalski, D. T., Łosińska, K., Prończuk, M., Markiel, A., Markowski, J., Pilch, J., Motowidło, J., Stastny, P., & Maszczyk, A. (2025b). Spasticity and pain and the improvement of muscle strength in athletes with partial spinal cord injury. *Baltic Journal of Health and Physical Activity*, 17(1), 10. 10.29359/BJHPA.17.1.10

[ref50] Skalski D. T., Pronczuk M., Losinska K., Lulinska E., Motowidlo J., Zurowska-Cegielska J., Bartosz-Jefferies M, & Maszczyk A. (2025c). Electromyography normalization and assessment methods for muscle activity: A systematic review and meta-analysis. *Baltic Journal of Health and Physical Activity*, 17(3), 4. 10.29359/BJHPA.17.3.04

[ref51] Skalski, D., Łosińska, K., Prończuk, M., Tyrała, F., Trybek, G., Cięszczyk, P., Kuliś, S., Maszczyk, A. & Pietraszewski, P. (2025d). Effects of real-time EEG neurofeedback training on cognitive, mental, and motor performance in elite athletes: a systematic review and meta-analysis. *Biomedical Human Kinetics*, 17(1), 249–260. 10.2478/bhk-2025-0024

[ref52] Stachenfeld, N. S. (2008). Acute effects of sodium ingestion on thirst and cardiovascular function. *Current Sports Medicine Reports*, 7(4 Suppl), S7–S13. 10.1249/JSR.0b013e31817f23fc 18843231 PMC2871322

[ref53] Stachenfeld, N. S. (2014). The interrelationship of research in the laboratory and the field to assess hydration status and determine mechanisms involved in water regulation during physical activity. *Sports Medicine*, 44(Suppl 1), S97–S104. 10.1007/s40279-014-0155-0 24791921 PMC4008811

[ref54] Takamata, A., Mack, G. W., Stachenfeld, N. S., & Nadel, E. R. (1995). Body temperature modification of osmotically induced vasopressin secretion and thirst in humans. *American Journal of Physiology: Regulatory, Integrative and Comparative Physiology*, 269(4), R874–R880. 10.1152/ajpregu.1995.269.4.R874 7485606

[ref55] Tucker, R., Rauch, L., Harley, Y. X. R., & Noakes, T. D. (2004). Impaired exercise performance in the heat is associated with an anticipatory reduction in skeletal muscle recruitment. *Pflügers Archiv: European Journal of Physiology*, 448(4), 422–430. 10.1007/s00424-004-1267-4 15138825

[ref56] Tucker, R., Marle, T., Lambert, E. V., & Noakes, T. D. (2006). The rate of heat storage mediates an anticipatory reduction in exercise intensity during cycling at a fixed rating of perceived exertion. *Journal of Physiology*, 574(Pt 3), 905–915. 10.1113/jphysiol.2005.101733 16497719 PMC1817748

[ref57] Verdel, N., Podlogar, T., Ciuha, U., Holmberg, H.-C., Debevec, T., & Supej, M. (2021). Reliability and validity of the CORE sensor to assess core body temperature during cycling exercise. *Sensors*, 21(17), 5932. 10.3390/s2117593 34502822 PMC8434645

[ref58] Wang, J., Lv, F., Yin, W., Gao, Z., Liu, H., Wang, Z., & Sun, J. (2023). The organum vasculosum of the lamina terminalis and subfornical organ: Regulation of thirst. *Frontiers in Neuroscience*, 17, 1223836. 10.3389/fnins.2023.1223836 37732311 PMC10507174

